# Generative replay underlies compositional inference in the hippocampal-prefrontal circuit

**DOI:** 10.1016/j.cell.2023.09.004

**Published:** 2023-10-26

**Authors:** Philipp Schwartenbeck, Alon Baram, Yunzhe Liu, Shirley Mark, Timothy Muller, Raymond Dolan, Matthew Botvinick, Zeb Kurth-Nelson, Timothy Behrens

**Affiliations:** 1 University of Tübingen, Tübingen, Germany; 2 Max Planck Institute for Biological Cybernetics, Tübingen, Baden-Württemberg, Germany; 3 Wellcome Trust Centre for Neuroimaging, University College London, London WC1N 3AR, UK; 4 Wellcome Centre for Integrative Neuroimaging, University of Oxford, John Radcliffe Hospital, Oxford OX3 9DU, UK; 5 State Key Laboratory of Cognitive Neuroscience and Learning, IDG/McGovern Institute for Brain Research, Beijing Normal University, Beijing, China; 6 Chinese Institute for Brain Research, Beijing, China; 7 Max Planck University College London Centre for Computational Psychiatry and Ageing Research, University College London, London, UK; 8 Institute of Neurology, University College London, London WC1N 3BG, UK; 9 Department of Psychiatry, Universitätsmedizin Berlin (Campus Charité Mitte), Berlin, Germany; 10 Google DeepMind, London, UK; 11 Gatsby Computational Neuroscience Unit, University College London, London, UK; 12 Sainsbury Wellcome Centre for Neural Circuits and Behaviour, UCL, London W1T 4JG, UK

**Keywords:** neural replay, prefrontal-hippocampal circuit, compositional inference, cognitive maps, flexible reasoning

## Abstract

Human reasoning depends on reusing pieces of information by putting them together in new ways. However, very little is known about how compositional computation is implemented in the brain. Here, we ask participants to solve a series of problems that each require constructing a whole from a set of elements. With fMRI, we find that representations of novel constructed objects in the frontal cortex and hippocampus are relational and compositional. With MEG, we find that replay assembles elements into compounds, with each replay sequence constituting a hypothesis about a possible configuration of elements. The content of sequences evolves as participants solve each puzzle, progressing from predictable to uncertain elements and gradually converging on the correct configuration. Together, these results suggest a computational bridge between apparently distinct functions of hippocampal-prefrontal circuitry and a role for generative replay in compositional inference and hypothesis testing.

## Highlights

• Human replay underlies ongoing inference about the composition of a stimulus
• Human replay becomes gradually refined to reflect the correct composition
• Representations of novel stimuli are relational and compositional
• These representations are found in human hippocampus and medial prefrontal cortex

## Graphical abstract

**Figure:**
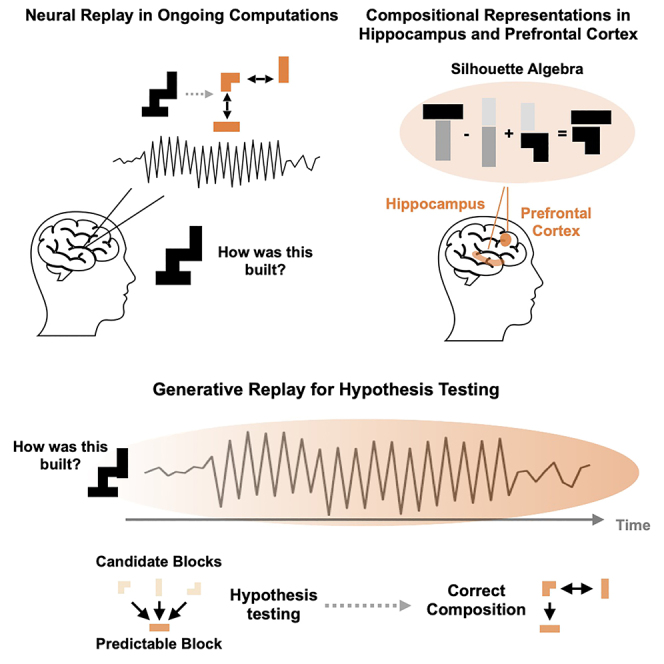

## Teaser

Generative neural replay underlies ongoing inference about the composition of a stimulus, resembling a hypothesis testing process. Relational and compositional representations in the hippocampal-prefrontal circuit underpinning such flexible inference generalize to novel and previously unseen problems.

## Introduction

Model-based reinforcement learning (RL) engages the hippocampus (HC) and prefrontal cortex (PFC)^1^^,^^2^^,^^3^^,^^4^ and makes plans using knowledge of transitions between states. However, unlike most RL problems studied in the laboratory, naturalistic inference problems are profoundly combinatorial. When a child builds a Lego tower out of 10 bricks, he or she is faced with more than 3.5 million possible brick orderings. It is not practical to enumerate the state space or learn about transitions in the vast product space. Nevertheless, model-based reasoning is a hallmark of human and other animal intelligence even at early stages of development.^5^^,^^6^ Solving this kind of task efficiently requires taking advantage of its compositionality.

One kind of compositionality is separating and recombining abstract relations and sensory specifics. For example, the concept of a brick being on top of another brick can be applied to any two bricks. Models built on this principal account for a wealth of neural data in the hippocampal formation and PFC.^7^^,^^8^^,^^9^

Another kind of compositionality is separating and recombining elements to make larger compounds. For example, brick A could be attached to brick B or to brick C. This kind of reasoning and inference is a constructive process.^10^^,^^11^ In generative models of scene understanding,^12^ embeddings of visual objects generalize across different scenes. Such representations enable agents to engage in flexible compositional reasoning and inference, a hallmark of “combinatorial generalization” and a potential path for agents to make “infinite use of finite means.”^13^^,^^14^ We use the term *flexible inference* to refer to this process of combining task knowledge in various potentially novel and unseen ways. We contrast such processes with other tasks that require inference over a much more restricted latent space, such as inferring whether an animal is currently in context A or B to determine a particular choice rule.

Less is known about the neural basis of this type of assembly,^5^ but it is also thought to engage the hippocampal formation and medial prefrontal cortex (mPFC).^15^^,^^16^^,^^17^ This is perhaps most strikingly demonstrated in natural scene perception tasks, such as imagining novel viewpoints, in which HC and mPFC are causally engaged.^18^^,^^19^^,^^20^ The hippocampal formation is also critical for integrating visual information to “anchor” a cognitive map into a perceptual scene, based on input from higher-order visual regions.^21^^,^^22^^,^^23^ Literature on closely related tasks in mental rotation^24^^,^^25^ additionally highlights the role of the posterior parietal cortex in these operations.^26^^,^^27^

While such compositional representations might allow a compact representational form, they do not provide a mechanism for inferring the appropriate configuration (and therefore representation) of current experience. However, further consideration of known hippocampal phenomena suggests a candidate substrate for this inference. In neural replay, sequences of cellular ensembles encoding external states of the environment are (re-)activated in time-compressed form.^28^^,^^29^ Critically, the external states that are activated are non-local. Replay events have been suggested as a substrate not only for memory consolidation but also for evaluating plans of the future.^30^^,^^31^ One possible unifying account of these apparently disparate ideas casts replay as a mechanism for learning and sampling from generative models of the world, often referred to as *generative replay*.^32^^,^^33^ If true, such an account suggests that replay might be involved in online computations to *understand the present*. Thus, when constrained by sensory data, such generative models are an essential part of any inferential process.^34^^,^^35^

To test these ideas, we designed two studies to investigate the neural representations and mechanisms that enable flexible compositional reasoning. We found compositional representations in the mPFC and anterior HC, reflecting the generalizable embedding of sensory building blocks in their relational configurations. Further, we show that generative sequences of hypothetical constructions are played as the subject understands the scene. These sequences resemble hypothesis tests during compositional inference.

## Results

### Subjects solve a compositional construction problem

**Figure 1:**
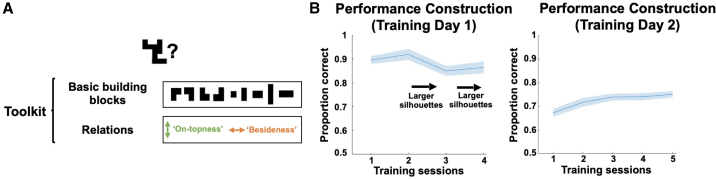
Paradigm and behavioral training (A) On 2 consecutive days, subjects were trained on nine basic building blocks, which could be flexibly combined by placing one building block on top of (below) or beside (left or right) another building block. (B) The complexity of the target silhouettes increased gradually, and subjects achieved overall high performance both in the actual construction (left) and determination of present building blocks under time pressure (right). Shaded colored areas reflect standard errors. See also Figure S1.

In a first study, we trained 30 human subjects on the task contingencies of constructing silhouettes out of a set of building blocks over 2 consecutive days (Figure 1A). Subjects learned that they had nine basic building blocks available that could be combined by placing them on top of or beside one another, without regard for the physical stability of the constructed object (similar to playing two-dimensional Lego or Tangram, see Figure 1A). Every building block could only be used once. The resultant construction task has considerable computational complexity: there are at least 6 × 10^12^ ways of connecting the nine building blocks, implying that an exhaustive state space representation including all possible configurations is computationally intractable.

**Figure S1:**
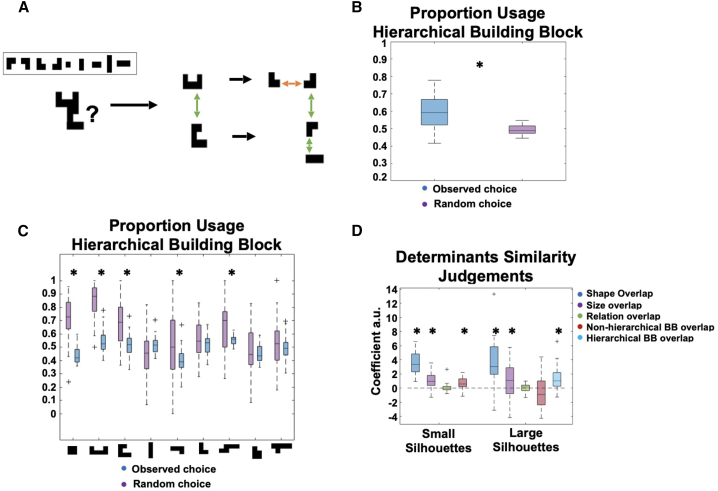
Behavioral effects, related to Figure 1 (A) We included an implicit hierarchical structure in the task, such that large silhouettes could often be decomposed into hierarchical building blocks. These hierarchical building blocks were never introduced explicitly but allowed for a more efficient construction of larger objects once learned. (B) Subjects displayed a preference for such “hierarchical chunking,” such that on the second training day they used a hierarchical building block configuration to construct larger silhouettes more often than predicted by chance. (C) Preferences for hierarchical chunking for the individual hierarchical building blocks. (D) At the end of the experiment, subjects completed a behavioral questionnaire to indicate similarity judgments between silhouettes. We found these similarity judgments were influenced by visual similarity, namely shape (pixel) and size overlap, and also by “construction similarity,” namely by the overlap of (basic/hierarchical) building blocks (BBs) across (small/large) silhouettes.

Nevertheless, subjects managed to solve the most basic version of the task immediately. Figure 1B shows participants’ performance on the 2 days of training. On day 1 (left), subjects had to construct silhouettes that increased in complexity and immediately achieved high overall performance (mean proportion correct: 0.89, SD = 0.09), which remained stable over the subsequent sessions. On day 2 (right), subjects were presented with a target silhouette and had to select the correct building blocks to construct this silhouette within a short amount of time. Again, subjects displayed high performance that gradually increased over time. To test whether generalizable inference extends across hierarchical levels, we added a hierarchical structure to the task (Figure S1A), such that larger silhouettes could often be decomposed into smaller recurring chunks. We found behavioral evidence that subjects made use of the hierarchical structure during flexible construction (Figures S1B and S1C). We did not detect neural representations reflecting this hierarchy, and consequently the following fMRI analyses collapsed across hierarchies. Using a behavioral similarity paradigm, we found that subjects processed silhouettes both in terms of their visual as well as compositional properties (see STAR Methods and Figure S1D).

Taken together, these behavioral data suggest that subjects quickly achieved a successful representation of the generalizable task structure during behavioral training, despite the considerable computational complexity of the task. This motivates the question about the neural basis that underlies such flexible and generalizable inference.

### Visual inference signals underlying the flexible construction of silhouettes

**Figure S2:**
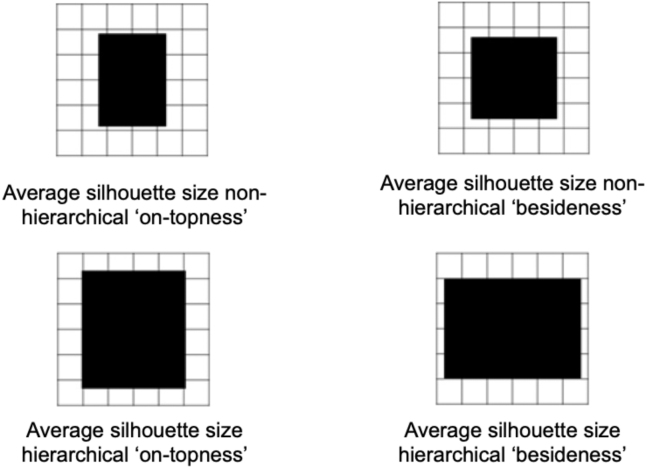
Stimulus properties, related to STAR Methods Average size for non-hierarchical (top row) and hierarchical (bottom row) compounds built by placing one (basic or hierarchical) building block on top of (left column) or beside (right column) another (basic or hierarchical) building block.

**Figure 2:**
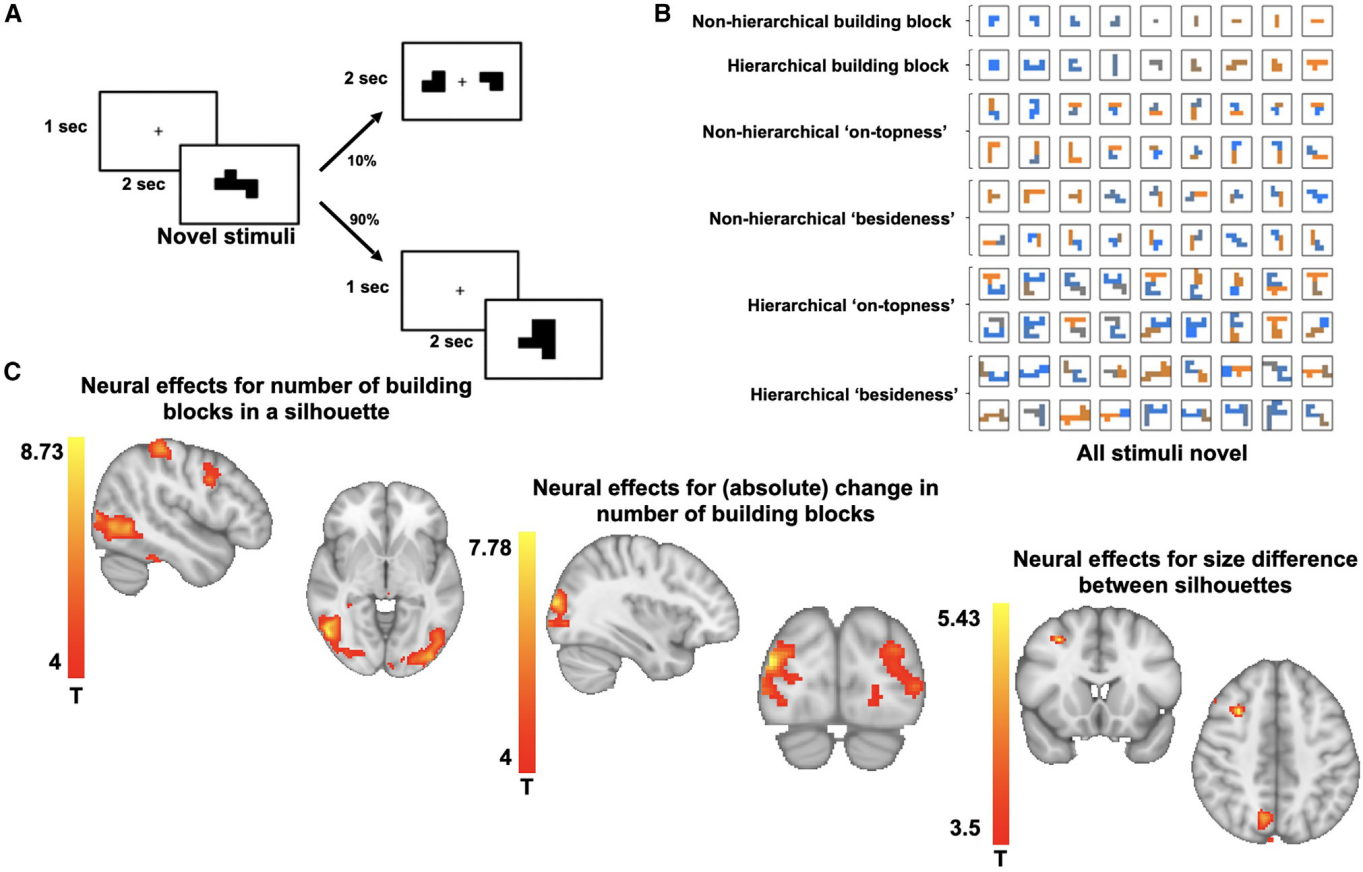
Neural effects of visual processing (A) In the fMRI-scanner, subjects saw a silhouette for a short period of time and were instructed to infer a plan for the construction of that silhouette. Sometimes trials were followed by a catch trial, in which subjects had to indicate whether blocks were part of the construction of the previous silhouette. (B) In the scanner, subjects received (known) basic building blocks (first row), hierarchical building blocks (second row), or novel and previously unseen compounds as construction trials. The novel compound silhouettes were either built with two basic building blocks on top of each other (third and fourth row) or beside each other (fifth and sixth row) or with two hierarchical building blocks on top (seventh and eighth) or beside (ninth and tenth) each other. (C) We found that activity in the lateral occipital, superior parietal, and precentral gyrus covaried with the number of elements in a compound, providing an approximation to construction difficulty (left). We also found effects for (absolute) changes in the number of elements between consecutive silhouettes in the lateral occipital cortex (middle). We did not detect any significant effects for differences in visual shape (pixels) but detected effects in superior parietal and frontal cortex reflecting differences in size between the individual silhouettes.

After two sessions of training on separate days, we measured the neural representations underlying the flexible construction of silhouettes using fMRI. In the scanner, subjects saw silhouettes for a short period of time and were instructed to infer a plan to construct these silhouettes. To ensure that subjects actively engaged in the mental construction, 10% of all trials were catch trials (see STAR Methods). Despite the challenging nature of the task and the short time period for the construction and probe trials, subjects achieved above chance accuracy in these probe trials (mean reaction time: 1,305 ms, proportion correct: 0.65). We selected silhouettes whose construction features (particular building blocks in particular relational positions) and visual features (such as the size or visual shape of the silhouette) were de-correlated (see Figure S2). In the scanner, trials included (basic and hierarchical) building blocks as well as compound silhouettes. Critically, these compounds were novel silhouettes that had never been experienced during training (Figure 2B).

Initially, we probed for effects of basic (visual) processing during the mental construction of a silhouette (Figure 2C). We found strong effects for activity in the lateral occipital cortex (peak Montreal Neurological Institute [MNI] [52 −66 −4], t_peak_ = 8.73), superior parietal cortex (BA7, peak MNI [−22 −74 58], t_peak_ = 8.04), and precentral gyrus (peak MNI [48 6 34], t_peak_ = 6.37, peak MNI [−48 −2 34], t_peak_ = 5.08) that covaried with the number of basic building blocks in a given silhouette, serving as an approximation to task difficulty and engagement in the construction process (left). We also observed strong effects in the lateral occipital cortex for (absolute) changes in the number of building blocks between consecutive silhouettes (peak MNI [−26 −94 14], t_peak_ = 6.08, peak MNI [38 −88 16], t_peak_ = 7.78). In this and all following imaging analyses, we controlled for shape (pixel) and size overlap effects as potential visual confounds in our analyses. We did not detect any significant effects for differences in pixel overlap between visual silhouettes, but we found effects for size differences in the superior parietal cortex (peak MNI [10 −68 46], t_peak_ = 4.74) and medial frontal gyrus (peak MNI [30 16 46], t_peak_ = 4.74). All effects are cluster-corrected at p < 0.001. This suggests that the component building blocks are reflected in visual activity over and above the basic visual properties of the silhouette.

### Compositional and relational neural representations in the medial prefrontal cortex and hippocampal formation

Our task design allowed us to go further than probing the effects of visual processing and to investigate neural representations that facilitate the internal construction of the object from its component parts—an instance of compositional reasoning.^36^^,^^37^^,^^38^ Specifically, our key hypothesis concerned the neural representations of building blocks in specific relational configurations that can be generalized across different stimuli, such as knowing what it means for an object to be on top of other objects. Such a representation implies neural patterns that encode specific *conjunctions* of a given building block in a given relational position, for example, a building block on top of but not below another building block. Such conjunctive representations can be flexibly combined, such as adding $\frac{W}{}$ (W on top of something) to $\frac{}{X}$ (X below something), together providing a neural code for the composed object $\frac{W}{X}$ (note that these are spatial relations of blocks, not fractions).

**Figure 3:**
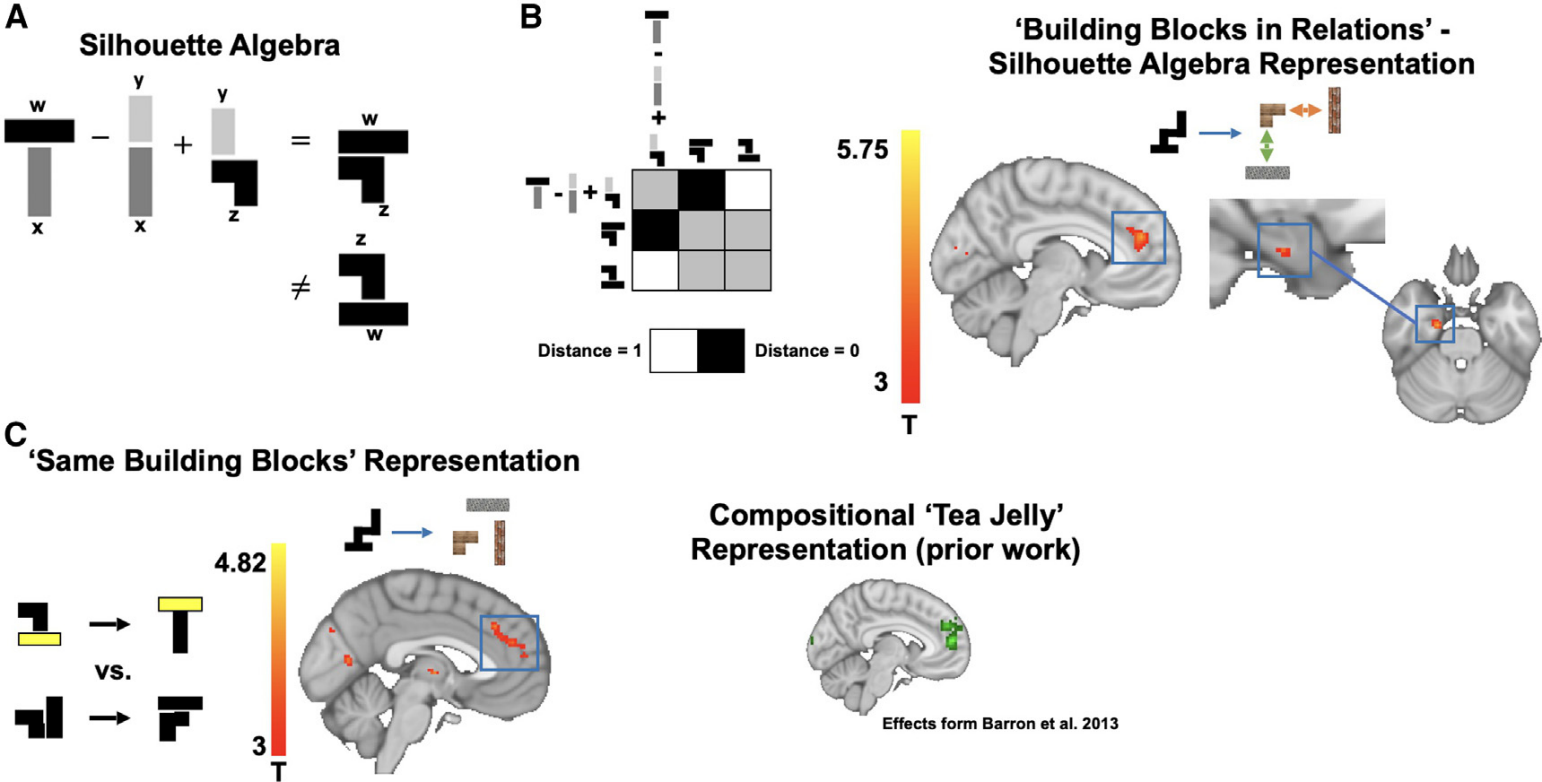
Construction inference is relational and compositional (A) We designed an analysis to test for generalizable representations of individual building blocks in specific relational positions by performing algebraic operations with neural representations for different silhouettes. For given building blocks WXYZ, the silhouette algebra predicts that $\frac{W}{X}-\frac{Y}{X}+\frac{Y}{Z}=\frac{W}{Z}$ (note these are spatial relations of blocks, not fractions). Under a conjunctive representation, the algebraic term on the left should be predictive of the actual silhouette with building block W on top of building block Z (target) but not of a silhouette with building block Z on top of building block W (reference). (B) Left: we tested in which brain regions such algebraic terms are predictive of target silhouettes but not reference silhouettes, using RSA, where we assessed whether the distance (defined as 1-correlation between activity patterns) between algebraic terms and target silhouettes is smaller than between algebraic terms and reference silhouettes (see STAR Methods for details). Right: we found significant effects in mPFC and the anterior hippocampus, extending into the entorhinal cortex, suggestive of a conjunctive representation of building blocks in specific relational positions. (C) Using repetition suppression, we probed the neural representations encoding for individual building blocks in a given construction problem, using an approach we reported in previous work when subjects had to imagine and evaluate novel food items.^39^ In regions encoding such representations, we expect higher suppression for transitions between silhouettes that share building blocks than transitions of silhouettes that use different building blocks. As predicted, we found the strongest suppression effects in the medial prefrontal cortex (red), highly overlapping with representations underlying the construction and evaluation of novel food items reported earlier (green, Barron et al. ^39^; Figure 2C).

**Figure S3:**
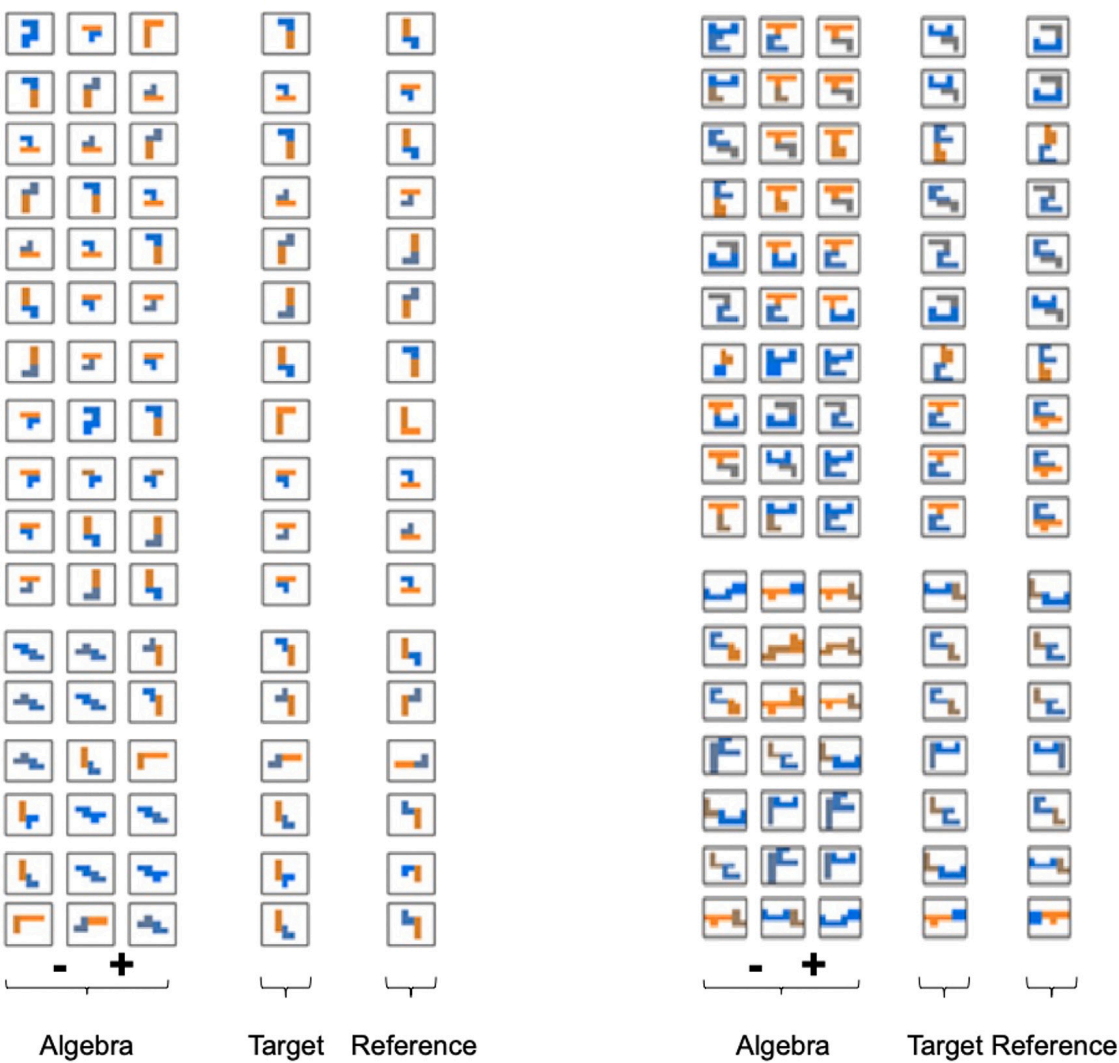
Stimulus properties silhouette algebra, related to STAR Methods All non-hierarchical (left) and hierarchical (right) silhouette algebra trials.

Critically, this allows us to predict specific relational configurations of building blocks, using representations of other configurations in a “silhouette algebra,”^12^ as illustrated in Figure 3A (see Figure S3 for all trials). For example, given building blocks WXYZ, silhouette algebra says $\frac{W}{X}\;–\;\frac{Y}{X}+\frac{Y}{Z}=\frac{W}{Z}$. Notably, we can perfectly control for the building blocks that are used by asking that the left-hand side of the equation predicts $\frac{W}{Z}$ (target) but not $\frac{Z}{W}$ (reference) that uses the same blocks. Such a representation is *compositional*—it uses the same representations to encode the blocks in different constructed silhouettes—but also *conjunctive* as these representations differ depending on the relational position of the blocks.

We used searchlight representational similarity analysis (RSA)^40^ to assess whether terms on the left-hand side (silhouette algebra) are more similar to the target than the reference (Figure 3B; see STAR Methods). Across the two hierarchical levels, this silhouette algebra analysis predicted voxel-wise patterns in the mPFC (peak MNI [41 78 46], t_peak_= 4.67, p = 0.045 based on a cluster-mass family-wise error (I)-corrected whole-brain non-parametric permutation test, Figure 3B). We found that these effects are stronger for the non-hierarchical silhouettes alone in the mPFC (peak MNI [40 76 42], t_peak_ = 5.35, p = 0.022 based on a cluster-maIFWE-corrected whole-brain non-parametric permutation test) and also in the anterior HC, extending into the entorhinal cortex (peak MNI [29 51 21], t_peak_ = 4.69, p = 0.028 based on voxel-wise FWE-corrected non-parametric permutation test corrected for the bilateral hippocampal formation), as shown in Figure 3B (right). The latter finding is closely aligned and highly overlapping with recent findings that translations in abstract stimulus space can predict representations of stimuli in the anterior HC.^41^ We did not detect significant effects for a hierarchical silhouette algebra alone.

This suggests that the mPFC and anterior HC support representations that reflect the building blocks in their correct configuration. Previous work has also highlighted representations in the mPFC and HC when constructing novel items.^39^^,^^42^ Specifically, this work has found a critical involvement of the mPFC and HC in evaluating novel food items, such as “tea jelly” built out of “tea” and “jelly.” A central difference to the algebra analysis reported above is that a tea jelly neural code does not differentiate between different relational embeddings of the individual building blocks that were used to construct a specific food item.

We tested for an analogous tea jelly representation in our compositional construction task by disregarding the individual relational positions of individual building blocks in a compound and simply using the overlap of individual building blocks across silhouettes as a measure of similarity instead. This can be thought of as the “input” to a given construction problem, reflective of the relevant building blocks used in a given construction problem. To ensure consistency with earlier approaches, we employed cross-stimulus fMRI adaptation.^43^^,^^44^ Here, compositional tea jelly representations predict stronger suppression effects for silhouettes (i.e., transitions between silhouette-trials) that share the same compared with different building blocks.

Previous reports of such construction effects have been based on valuation tasks.^39^^,^^42^ By contrast, our task involves a construction paradigm with no valuation component. Despite these differences, we found repetition suppression effects for these “input” representations in overlapping neural structures, particularly in the mPFC (Figure 3C, red: compositional representations underlying construction, green: effects from Barron et al.^39^; Figure 2C; peak MNI coordinates voI-wise FWE-corrected and masked by effects of Barron et al.^39^: [2 52 16], t_peak_ = 3.91, p = 0.037).

### Temporal characteristics of compositional construction

Our fMRI data support the view that compositional inference engages the hippocampal-prefrontal circuitry. They further show that in these brain regions, representations for construction share similarities with those implicated in planning, evaluation,^39^^,^^42^ and spatial reasoning.^8^ This opens up the possibility that mechanisms known to represent possible *futures* in these planning contexts might also underlie hypothesis testing about possible *presents*.

One such mechanism is replay.^28^^,^^29^ In rodents solving spatial tasks, hippocampal cells signal the current location of the animal, but during rest^45^ and planning,^46^^,^^47^ they transiently signal sequences of remote locations. It is suggested that at least some of these events signal a roll-out of a model of the world to predict possible futures and enable choices.^30^^,^^31^^,^^48^ Recently, we and others have developed tools to measure such sequences non-invasively in humans, using magnetoencephalography (MEG),^49^^,^^50^ and shown that they share many properties with rodent replay. We therefore designed an MEG experiment to probe the temporal dynamics and potential mechanisms underlying generative and compositional inference.

**Figure 4:**
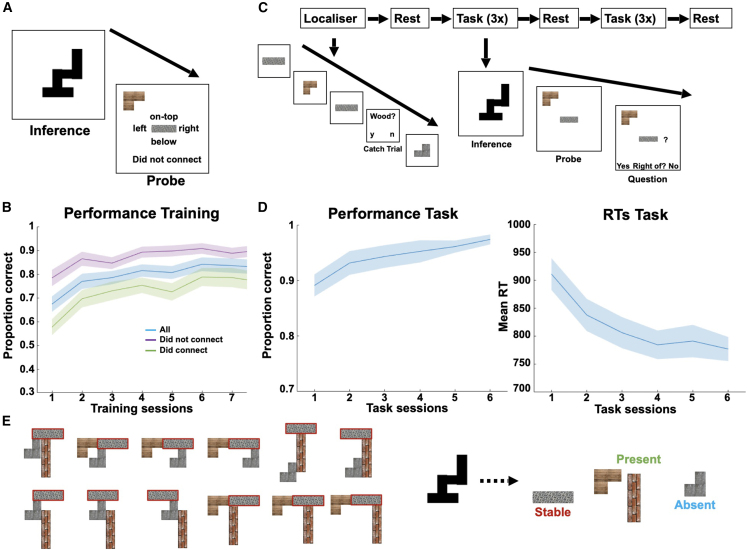
MEG task (A) The task consisted of an inference and probe phase. During inference, subjects were presented with a silhouette and had to infer its relational composition. During probe, subjects were presented with two building blocks and were asked to indicate the relation between these two building blocks in the previous silhouette, if any. (B) Subjects’ performance on the task improved over time. (C) The MEG experiment started with a functional localizer, where subjects observed individual building blocks with different textures (wood, concrete, steel, or bricks) on the screen. Intermittently, they received a probe question. The functional localizer was followed by a rest session, followed by three task sessions. The task was identical to training, except that we included an additional probe time window in which subjects were asked to infer the relation between two building blocks but could not yet indicate a response. The three task sessions were followed by another rest, followed by another three task sessions and a final rest session. (D) Subjects’ performance again improved over time, such that the proportion of correct responses increased, and reaction times decreased, with ongoing task experience. (E) In the MEG experiment, one building block was always present in every silhouette (*stable*, highlighted in red for an example stimulus set, see Figure S4 for all used stimuli), whereas two out of the remaining three had to be inferred (*present*) and one building block was *absent*. Shaded colored areas reflect standard errors.

Twenty human subjects were pre-trained on a construction task over 2 consecutive days (Figure 4A). This task was similar to the task used in the fMRI above but with two key differences. First, to optimize MEG decoding, we only had four building blocks and endowed each building block with a unique texture. This meant we could not have a hierarchical version of the task, which would require more than four blocks. Second, one of the four building blocks was present in every silhouette (“stable”). This was included to introduce asymmetry into possible plans, which allowed us to define the directionality of replay akin to forward and backward sequences.^50^^,^^51^ Here, these different directions translate into replay starting from the stable or present building blocks, as this asymmetry offers a natural way of constraining the hypothesis testing process.

After 2 days of training, in which performance gradually improved (Figure 4B), subjects participated in the MEG experiment (see Figure 4C). The MEG task started with a functional localizer to train binomial classifiers on the individual building blocks. This was followed by six task sessions in total, where subjects played the same task as during training on (initially) novel silhouettes. Every trial in the task had three phases: “inference,” “probe,” and “question.” During the inference phase, subjects were presented with a silhouette and had to infer its relational configuration. During the probe phase, subjects were presented with two building blocks out of the previous silhouette and had to find the relation between these blocks in the previous silhouette. In the question phase, subjects were probed about this relation. During the experiment, subjects displayed high accuracy in their performance (mean reaction time: 836 ms, proportion correct: 0.92) with a substantial improvement over time (Figure 4D).

**Figure 5:**
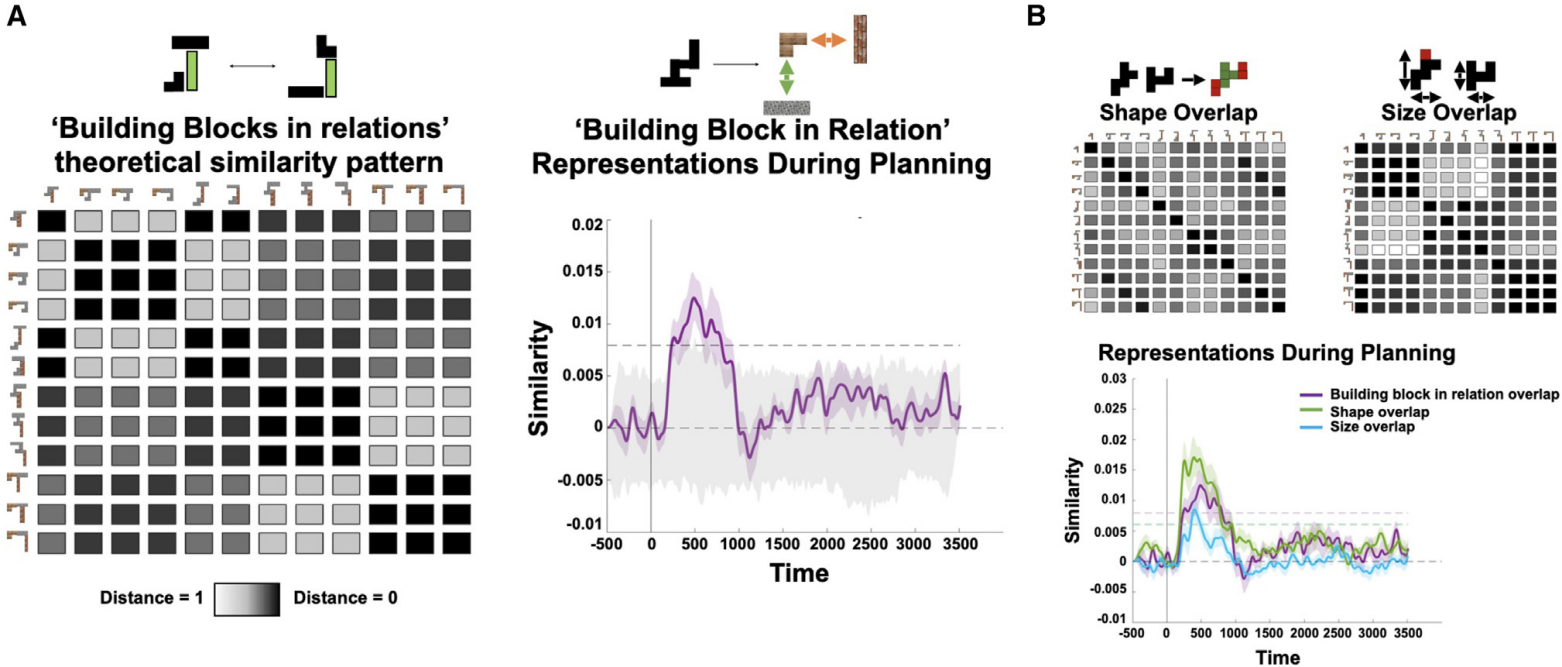
Conjunctive representations akin to the silhouette algebra from Figure 3B over time using RSA (A) Left: we defined a theoretical similarity reflecting the overlap of building blocks in specific relations across silhouettes, and we tested whether this similarity predicts empirical similarities of MEG sensor patterns across trials and time points. Right: we found a significant conjunctive representation, reflecting representations of silhouettes according to their constituent building blocks in specific relations, during a confined time window of 200–1,000 ms in the inference phase (significance assessed using a non-parametric permutation test, see STAR Methods for details). (B) We also found effects for shape (pixel) and size representational overlap during a similar time window during inference but with a slightly earlier onset. Note that the purple line in (A) and (B) are the same. Shaded colored areas reflect standard errors, and dotted lines reflect the statistical threshold obtained from a sign flip permutation test.

Our first analysis examined the representational content in the MEG sensors during the inference phase. As in the fMRI, we hypothesized that the MEG sensors would contain representations related not only to the visual appearance of the silhouette but also to the relational configuration of the inferred building blocks. Because of the always-present block, and because every silhouette has three building blocks, we could not perform the perfectly controlled algebraic analysis (where target and control consist of the same blocks in different relational positions). However, a proxy for this analysis is to test whether representational similarity across silhouettes is predicted by how many times the same building block appears in the same relational position in the two stimuli (Figure 4A). We performed RSA over time in the MEG data.^52^ For every trial and at any given time point, we assessed the empirical similarity of sensor representations. We regressed this empirical similarity matrix against predictions from the relational representation and from the visual similarity of the silhouettes (size and shape overlap, Figure 5B), acting as controls for the configural regressor. From 200 to 1,000 ms post-stimulus onset, there were strong independent effects of all three regressors in the MEG signal (particularly for shape and configural representations, Figure 5). While not as cleanly controlled as the fMRI data above, this suggests that the MEG data are also sensitive to both the visual and configural representations.

### Rapid neural sequences during compositional inference

Next, we asked whether replay plays a role in compositional inference. That is, whether hypothesized constructions were evident in rapid sequences in the MEG data. Recent work has shown that it is possible to measure replay in human MEG data.^49^^,^^50^ For example, recent studies have shown that when planning a trajectory through a discrete state space^49^ or resting after learning a sequence of pictures,^50^ individual items are replayed in sequences with a 40-ms time lag, reminiscent of sharp-wave ripple activity.^28^^,^^53^

**Figure 6:**
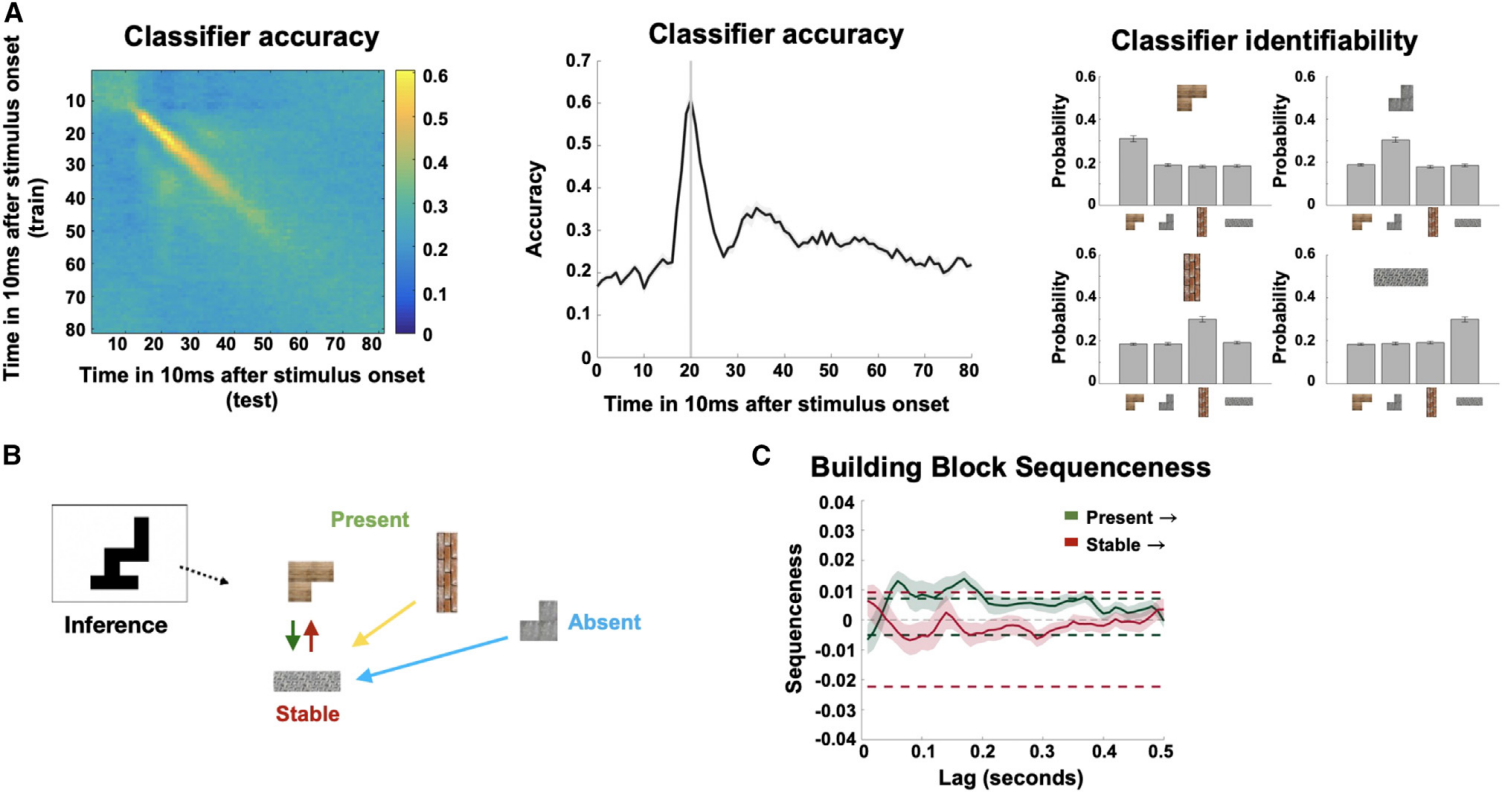
Neural replay in construction inference (A) We found peak decoding accuracy for building blocks in the localizer at 200 ms (left and middle) and high-class identifiability for the different building blocks for the classifiers trained at 200 ms (right). (B) In every silhouette, one building block was *stable* across silhouettes, two additional building blocks were *present*, and one building block was *absent*. This allowed us to define different types of sequences to (green) and from (red) the stable building block as well as between the present (purple) and absent (cyan) building blocks. (C) We investigated effects of neural replay for sequences starting either from the stable or the present building blocks. We found a short (non-significant) predominance of sequences starting from the stable building block for very early lags, followed by a predominance of sequences starting from the present building blocks at later lags with pronounced peaks at 60 and 170 ms. Shaded colored areas reflect standard errors. See also Figure S4.

We trained classifiers on building block identity, using the functional localizer data in the beginning of the experiment (see Figure S5 for sensor distributions of the classifier weights). In line with previous reports, we found that class identifiability peaked at 200 ms after stimulus onset (Figure 6A left and middle), and the classifiers displayed high specificity for identifying the correct building block when trained at that time (Figure 6A right; see STAR Methods). We used these classifiers in linear modeling^54^ to test whether reactivations of these representations occurred in specific (pairwise) orders and at specific time lags during inference.

**Figure S4:**
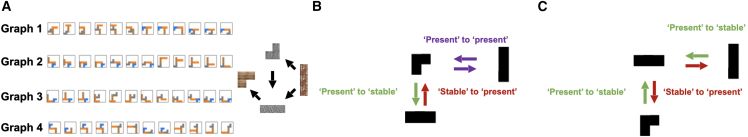
Types of building blocks in MEG task, related to Figures 6 and 7 and STAR Methods (A) Different stimulus sets used in the MEG task. Subjects were randomly assigned to one of these stimulus sets. (B) In half of the trials, the stable building block was not in the middle of the silhouette. (C) In the other half of trials, the stable building block was in the middle, such that there was no present to present building block connection.

Importantly, one (*stable*) building block was present in every silhouette. This meant that subjects could use this knowledge to constrain possible configurations (see below and Figure 4E). Each silhouette used two from the remaining three building blocks. On each trial, these two (*present*) building blocks were different and arranged in different configurations. This left out one (*absent*) building block in every trial that was not present in the silhouette (note the present and absent building blocks differed across silhouettes). In some trials, the stable block was connected to both present blocks. In other trials, the stable block was connected to one of the present blocks, and there was also a connection between the two present blocks (Figure 6B; see also Figures S4B and S4C).

**Figure S5:**
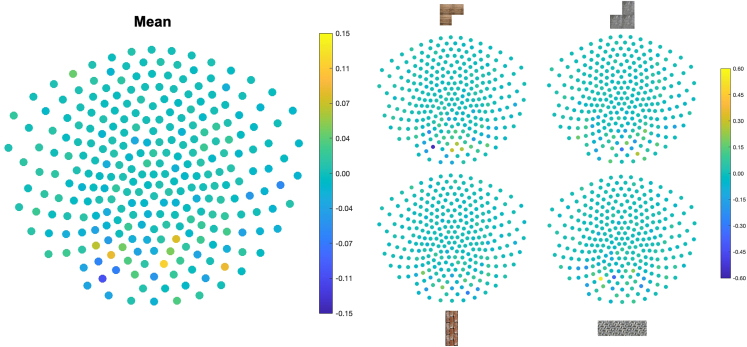
Sensor distribution, related to STAR Methods Sensor distribution of classifier weights for all (left) and individual (right) building blocks, trained on functional localizer data.

To establish whether neural sequences exist, we first examined sequenceness from stable and present blocks to their connected neighbors. We initially focused on the inference period, after elapse of the first 500 ms to avoid contamination from basic visual processes. We found little evidence for sequenceness starting from the *stable* building block but strong evidence of sequenceness starting from *present* building blocks (Figure 6C; see STAR Methods). Note that the x axis in Figure 6C is the temporal lag—the time difference *between representations that form a sequence*. This effect was significant at a broad range of temporal lags between 30 and 200 ms but had pronounced peaks at 60 and 170 ms—two time lags that correspond to reports in the previous human replay literature.^49^^,^^50^^,^^51^

**Figure 7:**
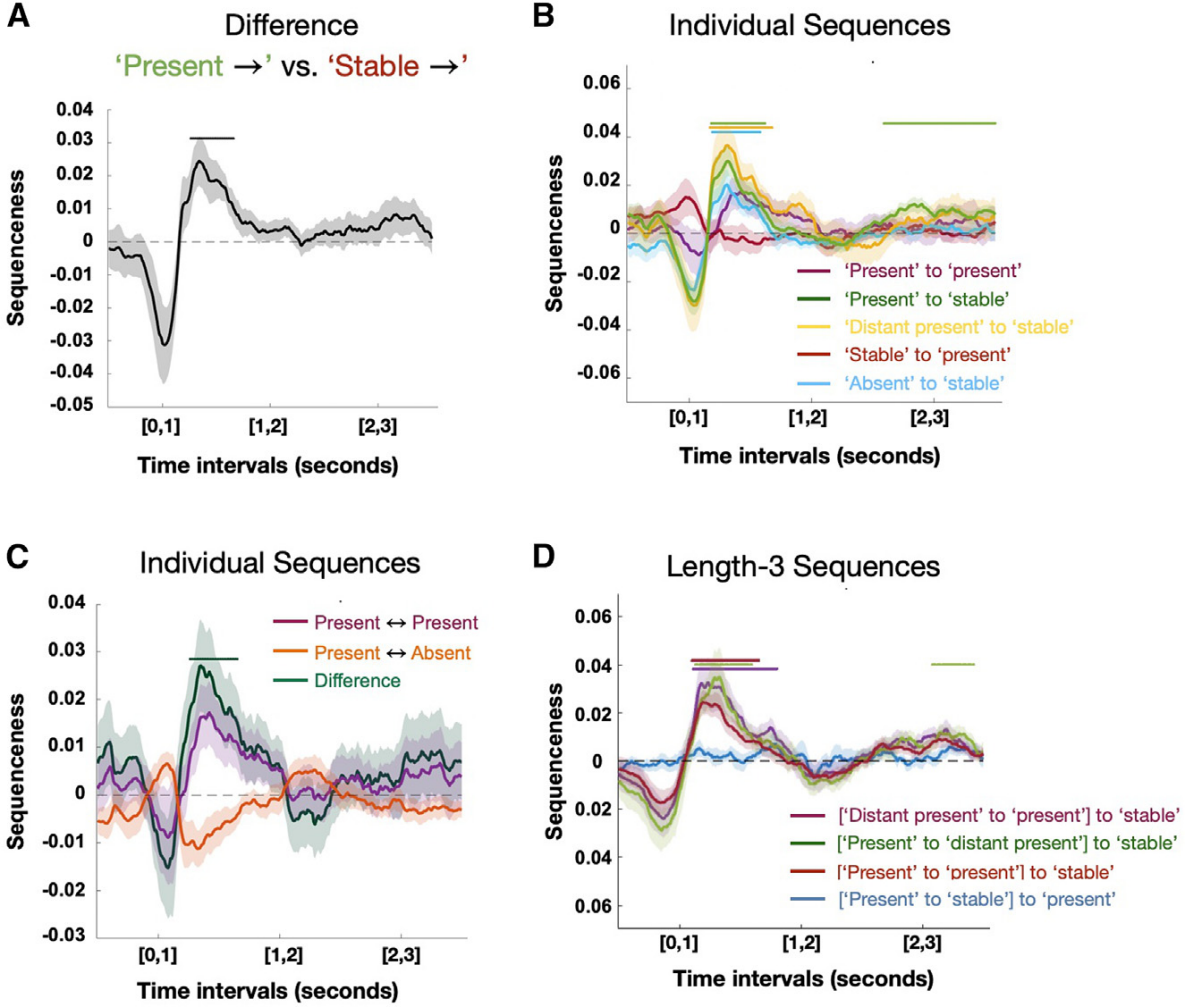
Generative neural replay underlying hypothesis testing over timescales of online computation (A) We investigated the difference between sequences starting either from the stable or the present building blocks for different time intervals of the inference period, and we found a brief early predominance of replay starting from the stable building block followed by a predominance of replay starting from the present building blocks (260–1,660 ms) during inference. (B) We assessed the individual contributions of the different types of neural replay to these differences and found an unspecific predominance of sequences from the present (180–1,620 ms), distant present (the present block that is unconnected to stable, 170–1,680 ms), and absent (190–1,580 ms) building blocks to the stable building block early during inference, as well as a specific effect from present to the stable building block late (1,590–3,500 ms) in inference shortly before subjects entered the decision phase of the task. (C) We found a selective predominance of replay between present building blocks over replay between present and absent building blocks in a time window between 260 and 1,650 ms. (D) We also tested for length-3 replay effects using this sliding window approach. This implies testing whether a specific transition between two building blocks predicts the transition to a third building block, while controlling for all possible length-2 and alternative length-3 transitions. Using this approach, we found significant effects for length-3 replay reflecting sequences from [present to present] to stable (100–1,650 ms), [distant present to present] to stable (110–1,800), and [present to distant present] to stable (130–1,590 and 2,080–3,420 ms). Shaded colored areas reflect standard errors. See also Figures S4, S6, and S7.

Understanding the computations executed in replay requires an examination of how the content of replay changed throughout the inference period. We therefore designed a moving-window analysis where we averaged over (10–200 ms) temporal lags. We computed this average sequenceness in 1-s windows centered at every 10 ms in the inference period. Hence, unlike Figure 6C, the x axes in Figures 7A–7D refer to the time *in the inference period*, not the temporal lag within the sequence. To demonstrate this method, Figure 7A shows the difference between sequences that start with *present* compared with those that start with *stable*. This is the same difference shown in Figure 6C but now measured at different times in the inference period. Here, the effect in Figure 6C is revealed as a significant cluster covering the time range 260–1,660 ms (cluster-corrected p < 0.05; see STAR Methods). This demonstrates sequences of building blocks during the inference period, which are constrained by the structure of the task in a way that guides relational inference, suggesting replay as a candidate mechanism of relational inference.

**Figure S6:**
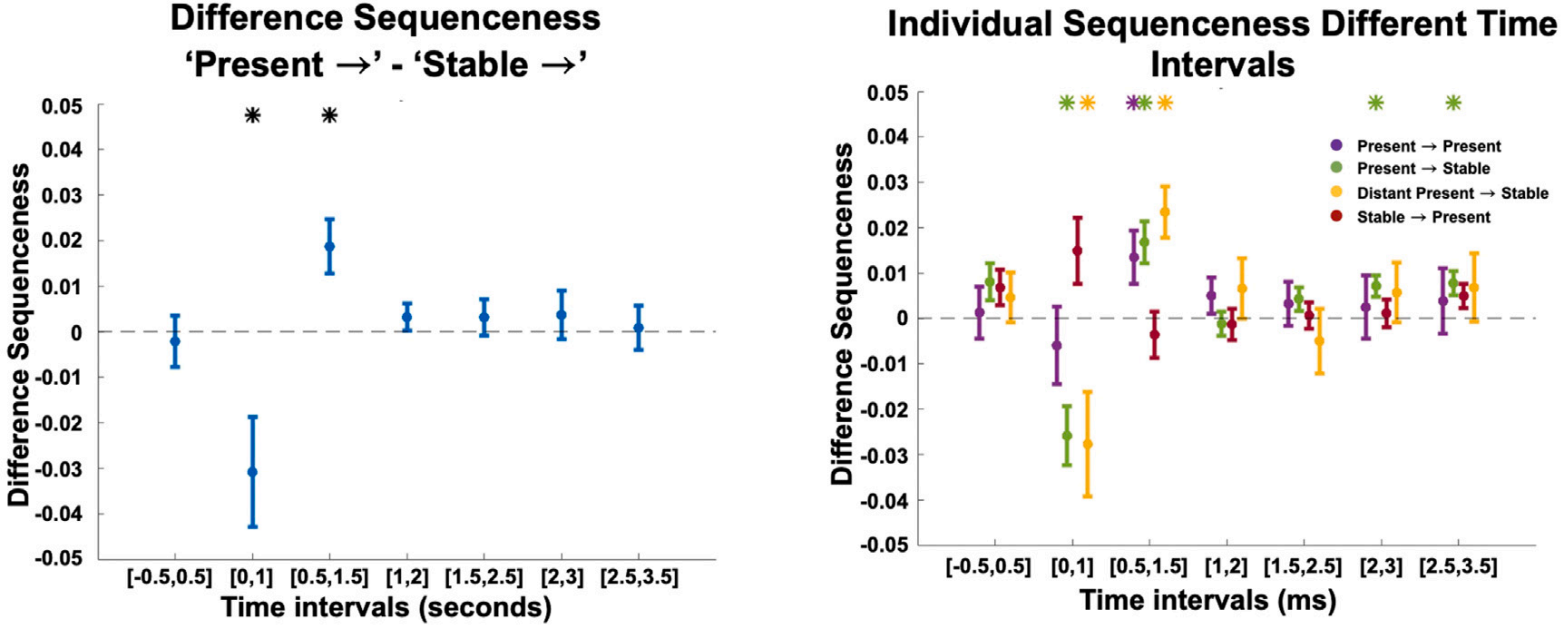
Discrete effects generative replay, related to Figure 7 We investigated the difference between sequences starting either from the stable or the present building blocks for different time intervals of the inference period, and we found an early predominance of replay starting from the stable building block (0–1,000 ms) followed by a predominance of replay starting from the present building blocks (500–1,500 ms) during inference (left). Assessing the individual contributions of the different types of neural replay to these differences, we found a marked decrease of sequences toward the stable building block early during inference (0–1,000 ms) followed by a predominance of sequences starting from the present building blocks (500–1,500 ms). We also found a specific predominance of sequences from present to the stable building block during intervals at the end of the inference period (2,000–3,000 and 2,500–3,500 ms) before subjects entered the decision phase of the task.

**Figure S7:**
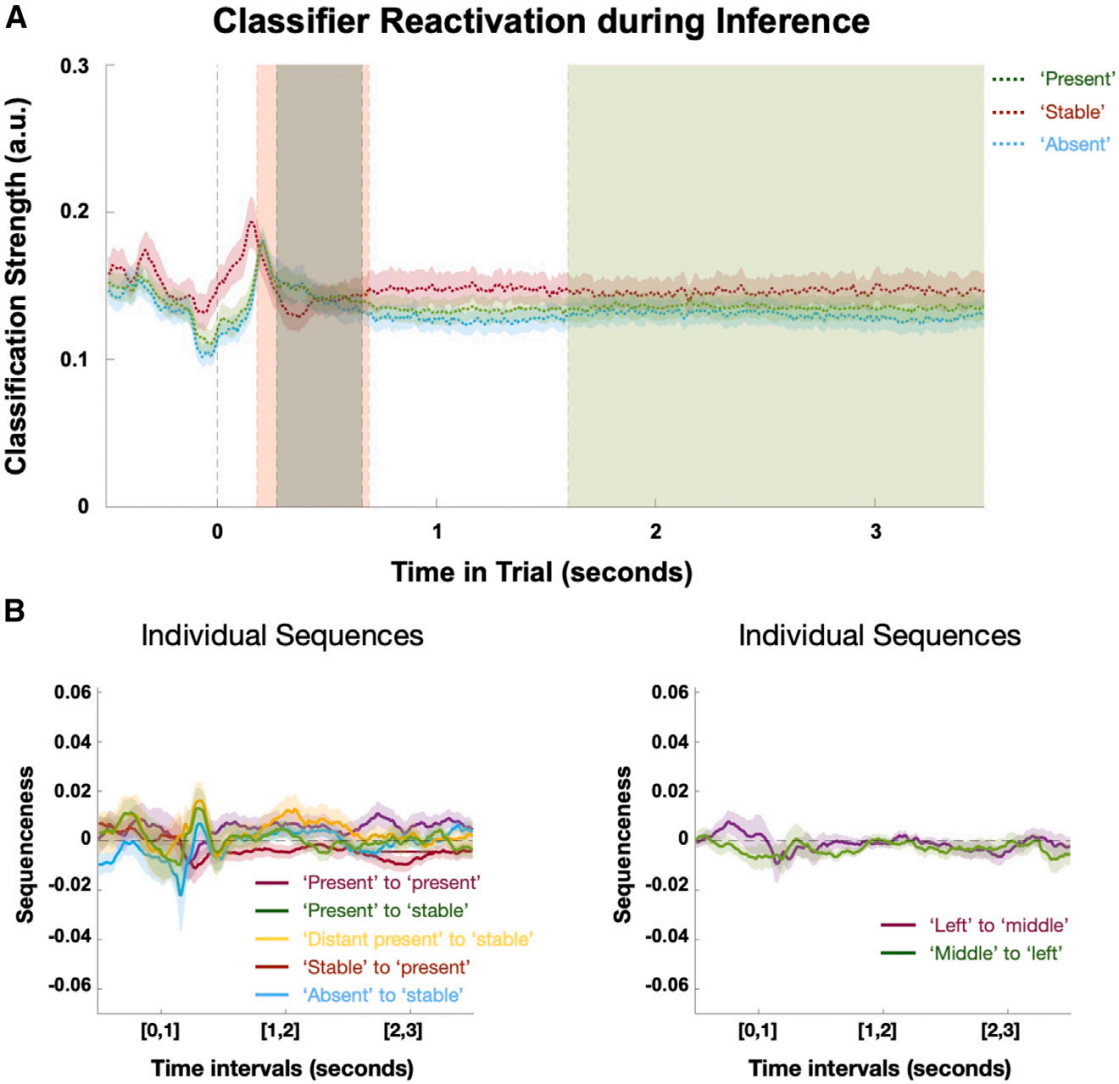
Classifier reactivation and replay effects during the probe phase, related to Figure 7 (A) We investigated the time course of the classifier reactivations for the stable, present, and absent building blocks averaged across trials. All reactivations peak shortly after stimulus (silhouette) onset, with the fully predictable stable building block representation peaking earlier. Overlaid are the time windows of the significant replay effects from Figures 6E and 6F (orange, significant effects for sequences from candidate building blocks to stable building block; dark green, significant difference between sequences between present and between absent and present building blocks; light green, significant effects for sequences from present to stable). (B) Left: while displaying a similar tendency as during the inference phase, we did not find significant replay effects for individual sequences analogous to Figure 7B during the probe phase. Right: we also did not detect replay for more task relevant information during the probe phase, such as the connection from the probe block in the upper left corner of the screen to the probe block in the middle or vice versa (right).

Also of note is a strong early negative effect (see also Figure S6). This might indicate a short very early period where replay emanates from the stable blocks but more likely is a confound: unlike all other blocks, the stable block can be predicted before the onset of the stimulus. Any later activation of other blocks (when the stimulus appears) will be measured as a forward sequence (as it comes after the pre-stimulus representation stable, see Figure S7). We therefore refrain from interpreting this peak here and in all later graphs.

### Replay as generative hypothesis construction

Our task structure confers a clear optimal strategy for sequential hypotheses, as the stable block constrains what solutions are actually possible. Subjects should begin the construction process by testing the stable block with all other candidate blocks. Once the neighbor(s) of the stable block is (are) resolved, the final step is to resolve any remaining connections between the two present blocks. Replay followed exactly this progression (Figures 7B and 7C). The earliest sequences in the inference period all proceeded toward the stable block and did not distinguish between present (180–1,620 ms), distant present (i.e., present but unconnected to stable, 170–1,680 ms), and absent (190–1,580 ms) blocks (blue and green lines in Figure 7B—note that these two analyses are orthogonal, and the blue, yellow, and green lines are independent measurements). Sequences that did not involve the stable blocks emerged later (260–1,650 ms) and *only involved the present blocks* (Figure 7C, purple/dark green line; note present-to-present sequences in purple are shown in Figures 7B and 7C to allow visual comparison of timings). Finally, at the end of the inference period, sequences to the stable block re-emerged, but these were only those involving the correct present blocks (1,590–3,500 ms, Figure 7B).

Note that Figures 7B and 7C show individual sequenceness effects, rather than differences between sequences (as in Figure 7A). This implies that a positive sequenceness effect implies higher reactivation probability of a certain building block after another block, such as the stable building block after the present building block. Likewise, a negative effect implies inhibition of a certain building block after another block, such as the absent building block after the present building block in Figure 7C.

In line with prior work,^50^ we also investigated evidence for length-3 replay between building blocks in a configuration. This implies testing whether a particular sequence between two building blocks (A to B) predicts the sequence to a third building block C, while controlling for all other possible length-3 and length-2 sequences. We indeed found evidence in favor of length-3 sequences, such that sequences between present building blocks were predictive of a subsequent representation of the stable building block. Specifically, we found significant effects for length-3 sequences from [“present” to “present”] to “stable” (100–1,650 ms), [“distant present” to “present”] to “stable” (110–1,800), and [“present” to “distant present”] to “stable” (130–1,590 and 2,080–3,420 ms).

Unlike the initial negative effect indicating sequences from stable to present that are likely caused by prior expectations about the presence of the stable building block, the subsequent sequenceness effects are not caused by purely representational differences of the present building blocks. In fact, when investigating the time courses of the classifiers for the stable, present, and absent building blocks, we see an early peak for the stable followed by (simultaneous) peaks for the present and absent building blocks before all classifiers return back to baseline (see Figure S7A). This implies that there is no specific temporal profile of those reactivation probabilities that could cause the above sequenceness effect, except for the initial negative effect in Figure 6E. Rather, these classification profiles are in line with our proposal of a hypothesis testing mechanism that simultaneously resolves uncertainty about the candidates, and our results indicate replay as a neural mechanism underlying these computations.

Taken together, these results indicate a role for replay in constrained hypothesis generation. Replay followed the optimal strategy for hypothesis generation, starting with unspecific sequences to the stable block, proceeding to infer connections between present blocks, and converging on sequences that only include correct blocks.

## Discussion

The hippocampal formation and PFC contribute to scene perception,^15^^,^^16^^,^^17^^,^^21^^,^^22^ the instantiation of a cognitive map during spatial and conceptual navigation,^55^^,^^56^^,^^57^^,^^58^^,^^59^ and model-based planning in RL.^1^^,^^2^^,^^3^^,^^4^ A key problem underlying these functions is learning an efficient representation of the state space and its relational structure that deals with the considerable complexity of naturalistic problems and enables generalization of knowledge to novel instances. We identified neural replay in the HC-PFC circuit as a candidate mechanism of generative hypothesis testing during such flexible inference.

Prior work has highlighted the importance of mPFC and hippocampal representations in the construction of novel compounds such as tea jelly out of known compounds such as tea and jelly.^39^^,^^42^ We detected representations in mPFC that reflect the use of a building blocks in a given compound irrespective of its relational embedding, highly overlapping with tea jelly representations reported earlier.^39^ Such representations are predicted under a “factorized code,”^7^^,^^8^ i.e., a representation of basic sensory building blocks that can be flexibly combined with structural knowledge to form novel conjunctive representations. It is an important challenge to understand how such structural or relational knowledge itself is represented efficiently, such that it can be flexibly inferred^60^ and adjusted to novel contexts, akin to a basis set for structural reasoning.^61^

Based on the involvement of the hippocampal-prefrontal circuit, we reasoned that generative replay provides a candidate mechanism for flexible construction. *Generative replay* refers to the hypothesis that replay reflects sampling from a generative model of the world to facilitate inference,^32^^,^^33^^,^^62^ enable generalization,^63^ and train a recognition model,^34^ providing a core mechanism for active hypothesis testing. In line with this hypothesis, we detected replay during constructive hypothesis testing. Replay sequences revealed an unspecific predominance for sequences in the direction of the fully predictable building block early during inference but a specific effect for replay from the correctly inferred present building blocks toward the predictable building block late during the inference period. We also detected more sequences linking correctly inferred present building blocks than sequences linking present and absent building blocks later during inference. These results suggest that generative replay may underlie hypothesis testing, with the results of this computation becoming increasingly refined as inference proceeds.

Our findings align with previous reports suggesting a role for replay during planning^49^ and learning^64^ in non-spatial problems, as well as evidence from recordings in animals suggesting that replay can explore novel trajectories.^30^^,^^48^ More broadly, our findings accord with notions that generative replay provides a mechanism for efficiently learning and sampling from a generative model of the world,^32^^,^^65^ in line with a crucial role of replay in planning^66^^,^^67^ and structure learning.^68^

In conclusion, we developed a paradigm to probe the neural mechanisms that underlie compositional reasoning. In close alignment with neural representations subserving both navigation and model-based RL, we found conjunctive representations in the hippocampal formation and PFC that flexibly generalize knowledge about relations between objects in a compound configuration. Further, we identified generative neural replay as a candidate mechanism underlying gradual hypothesis testing in construction problems. Together, these results provide insight into efficient neural representations that enable flexible generalization, supporting the hypothesis of a shared neural code underlying navigation, model-based RL, and compositional inference based on a cognitive map of task structure.

### Limitations of the study

In contrast to Barron et al.,^39^ we did not detect above threshold representations for individual building blocks in the HC, nor did we detect evidence for a purely relational code in the hippocampal formation or elsewhere. This negative result may be explained by the high degree of efficiency that participants obtained during 2 days of training prior to the scanning experiment. Participants may have strongly relied on a sequential mode of conjunctive processing (building block X below, building block Y on top, building block Z right of that, …) rather than an initially factorized representation that is subsequently conjoined (like Barron et al.^39^). Consequently, it would be of much interest to investigate the formation and change of the representations underlying these computations over the course of training.

We did not find significant replay effects during the probe phase (although the effects had similar tendencies), nor any other significant effects for task-based computations during the probe phase (Figure S7B). While we cannot draw conclusions from this null result, these effects are in line with our interpretation of replay being specifically involved in computations resolving uncertainty about a present configuration. Similar to prior work,^49^ we did not find a significant relationship between replay strength and performance, such as reaction times or the proportion of correct responses (see STAR Methods). One possible explanation for the lack of such a relationship might again be the over-training of participants, such that their performance was already close to ceiling when entering the scanner (see Figure 4D). This raises the intriguing question about a possible relationship between replay strength and performance during task learning.

Another limitation concerns the lack of a computational process model of generative and compositional inference as discussed here. Such a process model would be particularly impactful in making predictions for neural representations underlying more complicated compositional algebras. Owing to the lack of such a process model, we studied simpler and clearly defined compositions. Future work should bridge this gap and develop computational models that can solve such tasks with compositional representations to study the underlying neural representations in more complex environments.

By using non-invasive imaging techniques, we rely on indirect measures that limit our ability to make claims about the specific involvement of different brain regions or the origins of the generative replay signals. To understand the specific interplay of regions in the hippocampal formation and PFC underlying compositional inference, it would be crucial to obtain direct neural recordings. We believe that we have laid important groundwork for pursuing such investigations, based on our complex yet intuitive paradigm and our findings suggesting generative replay and a prefrontal-hippocampal involvement in compositional inference.

## STAR★Methods

### Key resources table

**Table:** 

REAGENT or RESOURCE	SOURCE	IDENTIFIER
**Deposited data**		

MEG data	This paper	https://github.com/schwartenbeckph/Generative-Replay https://doi.org/10.5281/zenodo.8303171

**Software and algorithms**		

MATLAB	Mathworks	https://www.mathworks.com/products/matlab.html
Temporally delayed linear modelling (TDLM)	Liu et al.^51^^,^^54^	https://github.com/YunzheLiu/TDLM
SPM	FIL Methods group, University College London (UCL)	https://www.fil.ion.ucl.ac.uk/spm Version: SPM 12
RSA Toolbox	Nili et al.^69^	http://www.mrc-cbu.cam.ac.uk/ methods-and-resources/toolboxes/
Custom code and algorithms	This paper	https://github.com/schwartenbeckph/Generative-Replay https://doi.org/10.5281/zenodo.8303171

**Other**		

Human healthy participants recruited from local area	This paper	N/A
Neural recordings and amplifier	Whole Brain CTF MEG 275 System	https://www.ctf.com/
3 Tesla Magnetom MRI scanner	Siemens	N/A

### Resource availability

#### Lead contact

Requests for further information should be directed to and will be fulfilled by the lead contact, Philipp Schwartenbeck; pschwartenbeck@gmail.com

#### Materials availability

The study did not generate new unique reagents.

### Experimental model and study participant details

#### fMRI task

30 subjects (25 females, mean age: 22.9, range: 19-33) participated in behavioural training and a subsequent fMRI experiment. Additionally, we scanned two pilot subjects and one subject did not participate in the fMRI part of the experiment after the behavioural training. All subjects were recruited from the UCL psychology subject pool, had no history of neurological or psychiatric illness and had normal or corrected-to normal vision. All subjects gave written informed consent and the study was approved by the UCL ethics committee (ethics code: 11235/001).

#### MEG task

20 subjects (15 females, mean age: 25.4, range: 20-36) participated in the behavioural training and subsequent MEG experiment. We scanned two pilot subjects prior to the experiment and one subject had to be excluded from the analysis due to impaired vision. All subjects were recruited from the UCL psychology subject pool, had no history of neurological or psychiatric illness and had normal or corrected-to normal vision. All subjects gave written informed consent and the study was approved by the UCL ethics committee (ethics code: 11235/001).

### Method details

#### fMRI task

##### Training and fMRI task

Subjects completed two tasks during behavioural training on two consecutive days. Initially, subjects completed four sessions (50 trials each) of the construction task on the first day of training. Subjects were instructed that in this and every subsequent construction task, every building block could only be used once for a given silhouette and that they had to find a solution using the minimum number of building blocks, allowing precise experimental control over the correct solutions that subjects had to infer. In this version of the training, there was no time restriction, and subjects familiarised themselves with the task contingencies. In every trial, subjects were presented with the nine basic building blocks at the top of the screen and saw a target silhouette at the bottom left. They then had to construct the target silhouette by selecting the correct building blocks and moving them around on the screen using a computer keyboard, being instructed that a construction would only be marked correct if they found a solution with the minimum number of elements. Further, every building block could only ever be used once. Silhouettes increased in size and complexity over the course of training. On the second day of training, subjects had to solve five sessions (70 trials each) of a similar task, but this time only select the correct building blocks without actually constructing the silhouette. This version of training had a time restriction, such that subjects had 6 seconds to infer a construction plan for a given silhouette followed by 6 seconds to select the correct building blocks. This task was designed to train subjects on the rapid mental construction of a silhouette that was required in the fMRI. In both tasks, subjects received feedback at the end of a trial indicating whether the construction or selection was correct, and they received 3 pence per correct answer in the second version of the training task.

To test whether flexible constructive inference extends across different hierarchical levels, we added an additional layer to the task. With ongoing experience, subjects could learn that larger silhouettes can often be decomposed into smaller recurring chunks, which are themselves built using two basic building blocks (Figure S1A). Thus, subjects were implicitly exposed to a set of ‘hierarchical’ building blocks, which facilitated an efficient decomposition of larger silhouettes. Analysis of participants’ behaviour on the second day of training provided evidence that when constructing larger silhouettes, subjects indeed chose ‘hierarchical’ building blocks more often than predicted by chance. Large silhouettes usually had more than one solution, not all of them required the usage of hierarchical building blocks. We probed how often participants relied on hierarchical chunks when constructing a silhouette compared to other available solutions that do not rely on such hierarchical building blocks (Figures S1B and S1C). We found that participants relied significantly more often on using such hierarchical chunks than a random agent (observed mean proportion of ‘hierarchical’ solutions: 0.6 (std=0.09), random mean proportion of ‘hierarchical’ solutions: 0.49 (std=0.03), t_mean difference_(30)=6.9499, p < 0.01). Note that this analysis does not rely on the analysis of building block selection order, which is contaminated by the spatial proximity of building blocks within hierarchical chunks. Rather, we focus on silhouettes that can be decomposed in different ways, and analyse how often participants found a solution based on a hierarchical compared to a non-hierarchical decomposition.

To impose hierarchical learning, we gradually introduced hierarchical building blocks into the training regime. In the first two training sessions of training day 1, subjects only had to construct silhouettes consisting of two building blocks. 27 of these 50 silhouettes in each session were hierarchical building blocks (3 trials per hierarchical building block) as illustrated in Figure 2B (second row). In the next two sessions of the construction task, subjects received larger silhouettes that often contained one or two hierarchical building blocks (third session: 18 hierarchical building blocks, 18 silhouettes with one hierarchical building block and one extra basic building block; fourth session: 18 silhouettes consisting of two hierarchical building blocks and 18 silhouettes with one hierarchical building block and one extra basic building block). Of the 70 trials in every session of the second training task, 24 where silhouettes that consisted of two hierarchical building blocks.

In the fMRI experiment, subjects had to solve a similar task to the second training task. Here, subjects were presented with a silhouette for 2 seconds and were tasked to mentally construct this silhouette. In 90% of trials, this was followed by a fixation cross for 1 second before presenting the next silhouette. In 10% of the trials, the silhouette was followed by a probe trial. In this probe trial, subjects were shown one or two basic building blocks and asked whether this/these building block/s can be used for the construction of the previous silhouette. Subjects had 2 seconds to respond ‘yes’ or ‘no’ via button press and received 20 pence for every correct answer. Every session in the scanner consisted of 288 trials in total, and subjects completed three sessions. In half of these trials, subjects were probed on a silhouette that either consisted of two basic or hierarchical building blocks (two repetitions per silhouette), combined with ‘on-topness’ or ‘besideness’ (i.e., one building block is on-top or left/right of another building block). In the other half of the trials, subjects were presented with one of the nine basic or hierarchical building blocks (eight repetitions per building block). In order to minimise effects of visual overlap of individual building blocks with silhouettes using these building blocks on the screen, the individual building blocks were presented at various locations throughout a session (twice at the top/bottom/left/right of the screen).

After the fMRI task, we assessed subjects’ individual similarity judgements about silhouettes that were presented in the scanner. To do so, subjects completed two sessions consisting of 120 trials in total, where they were presented with a target silhouette in the top middle of the screen and had to judge whether this target silhouette was more similar to a silhouette presented at the bottom left or right. Subjects had 6 seconds to make this judgement, followed by a 1 second inter-stimulus interval. In half of these 120 trials subjects were probed about silhouettes using basic building blocks and half of trials consisted of silhouettes using hierarchical building blocks. In the first of these two sessions subjects were instructed to focus on visual similarity, while in the second session subjects were instructed to focus on ‘construction similarity’ (‘which silhouette is more similar in terms of how you would construct them?’).

##### fMRI data acquisition

fMRI data was acquired on a 3T Siemens Prisma scanner using 32 channel head coil. Functional scans were collected using a T2^∗^-weighted echo-planar imaging (EPI) sequence with a multi-band acceleration factor of 4 (TR = 1.450 s, TE = 35 ms, flip angle = 70 degrees, voxel resolution of 2x2x2mm). A field map with dual echo-time images (TE1 = 10ms, TE2 = 12.46ms, whole-brain coverage, voxel size 2x2x2mm) was acquired to correct for geometric distortions due to susceptibility-induced field inhomogeneities. Structural scans were acquired using a T1-weighted MPRAGE sequence with 1x1x1mm voxel resolution. We discarded the first six volumes to allow for scanner equilibration.

#### MEG task

##### Training and task

Subjects completed two tasks during behavioural training. Initially, subjects completed two sessions of a construction task (50 trials each) of the same structure as in the beginning of the training for the fMRI task. In this task subjects only had four different building blocks available to construct silhouettes. After two sessions of the construction task, subjects were trained on a second version of the task that required them to make judgements about the relational configuration of given silhouettes. Subjects saw a silhouette for 6 seconds and had to infer the relational positions of individual building blocks in the silhouette. This was followed by a question screen lasting for 6 seconds, in which subjects were shown two building blocks and asked how they related to each other in the previous silhouette. Specifically, one of these building blocks was presented in the middle of the screen and the other at the top left of the screen, and subjects had to infer whether the building block in the top left was on-top, right, below, or left of the building block in the middle of the screen. They also had the option to indicate that the two building blocks did not connect in the previous silhouette. Subjects completed 3 sessions of this task on the first day and 5 sessions on the second day of training and received 5 pence for every correct answer.

After being trained on the task for two consecutive days, subjects participated in an MEG experiment on the day after the second day of training. In the scanner, subjects started with a resting session, in which subjects saw a fixation cross for 4 min and were instructed to maintain a state of wakeful rest. This was followed by a localiser screen for individual building blocks, which allowed us to train classifiers to decode individual building blocks from sensor activity (see below). Subjects completed two sessions in which each of the four building blocks was shown 25 times on the screen for 2 seconds. Subjects were instructed to focus on the building block identity, and particularly its texture (bricks, concrete, steel, or wood). To ensure that subjects actively engaged with the task, 10% of trials were followed by probe questions in which subjects had to indicate within 2 seconds via button press whether the previous building block was made of bricks/concrete/steel/wood. These two localiser sessions were followed by three task sessions (48 trials each). Subjects had to perform a task that was very similar to the training task where they had to infer the relation between two building blocks in a previous silhouette. In contrast to the training, the presented building blocks always connected to each other in the previous silhouette, such that the ‘did not connect’ option was removed in the MEG task. In this task, subjects saw a silhouette and had to infer a plan of its construction for 3.5 seconds, followed by a screen showing two building blocks out of the previous silhouette for 3.5 seconds, in which subjects had to infer how one building block related to the other in the previous silhouette. Finally, subjects saw a question screen for 1.5 seconds in which they were presented with one of four possible relations (on-top of, right of, below of, or left of) and had to indicate whether this was the relation they had inferred via button press (‘yes’ or ‘no’). In these question screens, probe relations could either be presented as text written at the bottom of the screen or via a question mark at the corresponding location (on-top, right, below, or left) of the building block presented in the middle of the screen to ensure that subjects process both the semantic meaning and the actual use in the construction of the inferred relation. The three task sessions were followed by another 4-min rest period, followed by another 3 task sessions and a final rest period.

##### MEG data acquisition

MEG was recorded continuously at 1200 samples/second using a whole-head 275-channel axial gradiometer system (CTF Omega, VSM MedTech), while participants sat upright in the scanner. Subjects indicated ‘yes’ and ‘no’ responses in both the functional localiser and MEG task using a scanner-compatible button box.

### Quantification and statistical analysis

#### fMRI task

We conducted a logistic regression to probe the influence of different silhouette characteristics on the similarity judgements. Specifically, for the small silhouettes we assessed the pixel overlap (defined as the maximum shape overlap of the two silhouettes across all possible translations along the presentation grid), size overlap, relational overlap (0/1 for whether the silhouettes were built with the same relation (ontopness/besideness) between the basic building blocks), and overlap of basic building blocks (BBs). For the large silhouettes we assessed the pixel overlap (defined as above), size overlap, relational overlap (0/1 for whether the silhouettes were built with the same relation (ontopness/besideness) between the *hierarchical* building blocks), overlap of basic building blocks, and overlap of hierarchical building blocks. We then computed the difference in those similarity measures between the left and right candidate silhouette, and defined a logistic regression to assess the predictability of these similarities for choosing the left candidate small silhouette:

$$p\left(choose\;left\right)=\frac{1}{1+e^{-\left(\beta_{0}+\beta_{1}\cdot Diff_{pixel}+\beta_{2}\cdot Diff_{size}+\beta_{3}\cdot Diff_{relation}+\beta_{4}\cdot Diff_{basic\;BBs}\right)}}$$

And for large silhouettes:

$$p\left(choose\;left\right)=\frac{1}{1+e^{-\left(\beta_{0}+\beta_{1}\cdot Diff_{pixel}+\beta_{2}\cdot Diff_{size}+\beta_{3}\cdot Diff_{relation}+\beta_{4}\cdot Diff_{basic\;BBs}+\beta_{5}\cdot Diff_{hierarchical\;BBs}\right)}}$$

Group level statistics was then obtained by performing a one-sample t-test on the resulting regression coefficients (see Figure S1D). Investigation of the resultant regression weights indicated that similarity judgements were guided by basic visual similarity, namely the shape (pixel, small silhouettes: $\beta_{mean}=3.46$ (std = 1.73), t=10.21, p<0.01, large silhouettes: $\beta_{mean}=3.73$ (std = 3.09), t=6.15, p<0.01) and size overlap (small silhouettes: $\beta_{mean}=1.16$ (std = 1.22), t=4.86, p<0.01, large silhouettes: $\beta_{mean}=1.06$ (std = 2.49), t=2.17, p=0.04) of the candidate silhouettes with the target silhouette. Importantly, however, we also found that the overlap of relevant building blocks (‘construction similarity’) accurately predicted similarity judgements. In small silhouettes that were compounds of two basic building blocks, the overlap of basic building blocks predicted subjects’ similarity judgements ($\beta_{mean}=0.62$ (std = 0.88), t=3.55, p<0.01), whereas in large silhouettes, the overlap of hierarchical building blocks was predictive of those judgements ($\beta_{mean}=1.39$ (std = 1.79), t=3.95, p<0.01) (Figure S1D).

#### Pre-processing

All pre-processing steps and subsequent imaging analyses were performed with SPM12 (Wellcome Trust Centre for Neuroimaging, http://www.fil.ion.ucl.ac.uk/spm). Functional images were corrected for signal bias and realigned to the first volume in the sequence using a six-parameter rigid body transformation to correct for motion. Images were then spatially normalised by warping subject-specific images to MNI (Montreal Neurological Institute) reference coordinates and smoothed using a 6-mm full-width at half maximum Gaussian kernel. The RSA-analysis was performed on unsmoothed data before smoothing the resulting contrast estimates (see below).

#### Repetition suppression analysis

We employed univariate repetition suppression analysis to test for compositional representations of individual building blocks within a silhouette. To do so, we modelled the onset of all objects on the screen as stick functions, and defined several parametrically modulated regressors of interest to control for potential confound variables. In total, we defined four control regressors that account for repetition suppression due to size or pixel non-overlap and change in the number of building blocks in a silhouette. Size non-overlap was defined as the absolute difference in height and width of silhouettes, and pixel non-overlap as the maximum proportion of overlap of pixels of two silhouettes relative to their full ‘pixel-size’ subtracted from 1. We also added the number of building blocks in a silhouette as an additional fourth control regressor. The effects for these control regressors are shown in Figure 2. Next, we defined three building block non-overlap regressors that account for compositional representations, i.e., representations of individual building blocks within a silhouette. We defined a regressor that reflected the proportion of non-overlap of the basic building blocks in a present silhouette with the basic building blocks of the previous silhouette (see supplementary information for an illustration). This regressor only had a unique value for small silhouettes that did not consist of hierarchical building blocks. This is because there was more than one solution of basic building blocks in large silhouettes (built with two hierarchical building blocks and four basic building blocks). Consequently, we split up this regressor that reflected the non-overlap of basic building blocks into trials with small (two basic building blocks) and large (two hierarchical building blocks, four basic building blocks) silhouettes. For large silhouette-trials that had more than one basic building block solution, we computed the average of building block non-overlap weighted by the different solutions for a given silhouette. In addition to those basic building block non-overlap regressors, we defined a hierarchical building block non-overlap regressor following the same logic but with hierarchical building blocks. Just as the regressor for basic building block non-overlap in small silhouettes, the regressor for hierarchical building block non-overlap (in large silhouettes) had only unique solutions. Finally, we defined three regressors that accounted for relational non-overlap. These regressors differentiated between trials of silhouettes that used the same or a different relational operation (putting a building block on-top or beside another building block) compared to the previous trial. We split this regressor into trials of small silhouette transitions (relational operation for basic building blocks), large silhouette transitions (relational operation for hierarchical building blocks), and transitions between small and large silhouettes. In order to make all these parametric regressors comparable, they were projected onto an interval ranging from -1 to 1.

Because of the sensitivity of the blood oxygen level-dependent (BOLD) signal to motion and physiological noise, all GLMs also included six motion regressors and their derivatives obtained during realignment, as well as 6 regressors for cardiac phase, 6 for respiratory phase and 2 for respiratory volume extracted with an in-house developed Matlab toolbox.^70^ Sessions were modelled separately within the GLMs.

To obtain the ‘tea jelly’-like compositional representation results (Figure 3C), we combined the effects for building block non-overlap with unique solutions, i.e., the basic building block non-overlap in small silhouettes and the hierarchical building block non-overlap.

#### ‘Silhouette algebra’ analysis

To test for the presence of a conjunctive code, we assessed the representational distance between algebra terms, target silhouettes and reference silhouettes. We performed volumetric searchlight RSA^40^ based on a GLM approach similar to Hunt et al.^71^ Effectively, we asked whether we can predict empirical distances (defined as a 1 - correlation metric) between activity patterns for algebra terms, target silhouettes, and reference silhouettes by theoretical distances predicted by a conjunctive code whilst controlling for visual confounds based on size and shape overlap. To do so, we first obtained individual coefficient estimates for all stimuli used in the fMRI task based on a first-level univariate GLM on unsmoothed data. We then defined searchlights across every voxel including the 100 cortical voxels with smallest geodesic distance from the central voxel.^72^ Coefficient estimates in every searchlight were pre-whitened. Both the searchlight definition and pre-whitening were based on adapted scripts from the RSA toolbox.^69^ We then defined a representational distance metric (defined as 1-correlation) between algebra terms, target and reference silhouettes. To do so, we first computed all possible algebra terms as shown in Figure S3. We then computed the distance to the respective target and reference silhouette, resulting in a sparse representational matrix of size [(algebra terms + target silhouettes + reference silhouettes) x 3 sessions] x [(algebra terms + target silhouettes + reference silhouettes) x 3 sessions]. To avoid any within-session similarity effects, we only computed and compared cross-session distances. Next, we tested whether this empirical representational distance matrix could be predicted by a theoretical representational distance that reflects a conjunctive code as shown in Figure 3B. For every searchlight, we computed a GLM to assess the prediction of the empirical representational distance matrix based on a conjunctive representation, whilst controlling for two additional theoretical distances based on the shape and size of the objects. This ensured that any shared variance between conjunctive and visual confound representations was removed, and resulted in a single conjunctive representation map per subject. These coefficient maps were smoothed using a 5mm FWHM kernel in line with a recent study based on a similar approach.^73^

#### Multiple comparison correction

To assess statistical significance on the group-level for conjunctive representations, we performed family-wise error (FWE) corrected sign-flip permutation tests^74^ using PALM^75^ either using a pre-defined ROI of the hippocampal formation based on the Juelich anatomical atlas^76^^,^^77^ or in a more exploratory whole-brain approach. Coefficient values of every subject were randomly multiplied by 1 or -1 based on the null-hypothesis that these coefficient values are symmetrically distributed around 0. To create a null distribution of the means this process was repeated 5000 times, and the true value was then compared to this null distribution. On the whole brain level, we used a maximum cluster mass statistic^74^ for FWE correction based on a cluster forming threshold of p < 0.001.

#### MEG task

##### MEG data preprocessing

The preprocessing protocol closely followed a recently published study.^50^ Data were resampled from 1200 to 100 Hz to improve signal to noise ratio and high-pass filtered at 0.5 Hz using a first-order IIR filter to remove slow drift. Subsequently, an ICA (FastICA, http://research.ics.aalto.fi/ica/fastica/) was performed to decompose the data into 150 temporally independent components and their corresponding sensor topographies. Artifact components were identified using automated inspection based on spatial topography, time course, kurtosis of the time course and frequency spectrum. Eye-blink artifacts can be identified based on high kurtosis (>20) and mains interference based on a low kurtosis and a frequency spectrum dominated by 50 Hz line noise. Based on these definitions, artifacts were rejected by subtracting them out of the data. Epoched data from the functional localiser and inference period during the MEG task was baseline-corrected by subtracting the mean sensor activity 100ms before stimulus onset from the data. Subsequent analyses were performed directly on the filtered, cleaned MEG signal in units of femtotesla on the whole-brain sensor level.

##### RSA

We performed GLM-based representational similarity analysis akin to our fMRI analysis. During the inference period of the task we obtained empirical representational similarity matrices for the different stimuli based on a similar approach reported in Luyckx et al.^52^ We defined a design matrix that specified the present silhouette at a given trial using one-hot vectors (12 one-hot vectors for 12 silhouettes in total) and one additional regressor to account for the mean activity. Using this design matrix, we then obtained sensor coefficients for each stimulus at any given time point, resulting in a sensor x stimulus x time-point matrix. The coefficients were pre-whitened using an adapted script from the RSA toolbox^69^ and then used to compute Pearson correlation coefficients between sensors, for every individual silhouette for every time-point. This resulted in a representational similarity matrix for silhouettes across time points for both the inference. Akin to the fMRI conjunctive code analysis, we then specified a GLM predicting these empirical similarities using different (z-scored) theoretical similarities across time (see Figure 5). During the inference period when subjects saw a silhouette on the screen, we defined conjunctive representations (building block in a specific relational position) as well as size and pixel overlap as theoretical representational similarities.

We defined a non-parametric permutation threshold to test for statistical significance. We repeated the above analyses with randomly shuffled predictor representational similarities 5000 times to create a null distribution of predictive coefficients. To define a significance threshold, we defined the 97.5^th^ and 2.5^th^ percentile of the range of the shuffled predictive coefficients as upper and lower significance thresholds.

##### Multivariate Decoding

Training of the classifiers and subsequent sequenceness analyses closely followed previously published approaches.^50^^,^^54^

We trained 4 Lasso-regularised regression models on the four different building block classes in the functional localiser data, using only sensors that were not rejected in all individual scanning sessions. This provided a decoding model based on a binomial classifier per building block, where we defined all trials in which a corresponding building block was present as positive examples and all other trials as negative examples. To decorrelate the classifiers we also included null data, defined as the sensor data from 500ms before stimulus onset. In line with previous work^49^^,^^50^ we found maximum decodability (defined as the highest probability output among all classifiers being assigned to the correct class) of individual building blocks 200ms after their onset and consequently trained the binomial classifiers on that time for the subsequent sequenceness analysis. In line with previous work,^49^ we used and L1 penalty of 0.006, encouraging sparsity in the sensor representations for the individual building blocks.

##### Sequenceness Measure

We defined sequenceness measures analogous to Liu et al.^50^^,^^51^^,^^54^. We used the trained classifiers to obtain class reactivation predictions for the independent inference data. The sequenceness measure is then obtained by applying a GLM approach at two levels. At the first level, we obtain empirical pairwise transitions or sequences between class reactivations by using a linear model to test whether certain stimulus reactivation patterns are predictive of other reactivation patterns at different time-lags (with a maximum lag of 500ms). This results in a 4x4 matrix of empirical building block transitions for every time-lag. Here, we did not include additional nuisance regressors to control for confounding effects, such as an alpha oscillation,^50^ since we applied this analysis to an active task-engagement period rather than a rest phase. At the second level, we then ask whether this pattern of empirical transitions at different time-lags is predicted by theoretical transition matrices whilst controlling for the mean and self-transitions. Note that a negative effect for sequences between building blocks (such as between present and absent building blocks) implies the inhibition of one after the other (i.e. a lower re-activation probability for absent after a present block and vice versa), not the inverse directionality.

We defined three different types of sequences of interest corresponding to three theoretical transition matrices: sequences from the ‘stable’ to the ‘present’ building blocks, sequences from the ‘present’ to the ‘stable’ building blocks and sequences between the ‘present’ building blocks (if applicable – in trials where the ‘stable’ building block was in the middle of the silhouette the two ‘present’ building blocks did not connect). We averaged the latter two to obtain the global effect of sequences starting from the ‘present’ building block as shown in Figure 6C.

Figure 6C shows the effects of the sequenceness analysis when applied to the full inference period except for the first 500ms to allow for basic visual processing. The obtained sequenceness effects were tested against control sequences, where one building block in the true theoretical transition matrix was replaced by the absent building block. This results in two alternative sequences for each of the three sequence types (‘present’ to ‘present’ if applicable) per trial (288 trials in total). We then treated the minimum and maximum of these control sequences across time-points (averaged over trials) as statistical bound, against which we compared the sequenceness for the true sequences.

Length-3 sequences were probed as described in Liu et al.^50^^,^^51^^,^^54^. This analysis relies on the same GLM approach, but now probes whether observed sequences of length 2 are predictive of the reactivation of a third building block, whilst controlling for shorter length transitions. We designed a GLM where we defined all possible pairwise sequences and individual building block re-activations as predictors for subsequent building block re-activations at different time-lags. We then probed the evidence for certain types of length-3 sequences, particularly for [‘present’ to ‘stable’] to ‘present’ and [‘present’ to ‘present’] to ‘stable’ across all trials, and [‘distant present’ to ‘present’] to ‘stable’ as well as [‘present’ to ‘distant present’] to ‘stable’ in trials where there was a ‘distant present’ building block (i.e., a present building block that was not directly connected to the stable block, in trials where the stable building block was not in the middle of the silhouette).

We also conducted a sliding-window approach to assess the prevalence of individual sequences over time. To do so, we defined sliding-windows of 1000ms with a step-size of 10ms (starting 500ms before stimulus onset and moving up to 3500ms after stimulus onset, resulting in 301 time windows in total, i.e. [-500,500],[-490,510],...,[2500,3500]). Within each window, we performed temporally delayed linear modelling for the different candidate sequences, and averaged the sequenceness effects for time-lags of 10-200ms. We have also included the same analysis using non-overlapping time-windows of length 500ms with a step-size of 500ms in the supplement, providing conceptually identical results.

Cluster-based statistics on these time windows was obtained similarly to Eldar et al.^78^ In the time-series, we assessed the length of consecutive time-points exceeding the critical t-value of +/-2.09 (two-sided P value of 0.05 for df=19). The data were then shuffled 10000 times by randomly multiplying half of the subjects’ time-series by -1 and obtaining the maximum length of consecutive time-points exceeding the critical t-value for that shuffle. We then defined the 95^th^ percentile of cluster lengths from the shuffled data as cluster-based significance threshold, against which we tested the original data.

We analysed the relationship between replay strength and performance. Both mean replay strength of the first peak in Figure 6E and mean replay strength of the second (present to stable) peak did not correlate significantly with mean reaction times (first peak: r=−0.23, p=0.32; second peak: r=−0.23, p=0.33) or proportion of correct responses (first peak: r=0.16, p=0.49; second peak: r=0.19, p=0.43) on the subject level. We also did not find a significant relationship between trial-by-trial replay strength of the first and second peak in Figure 6E and trial-by-trial reaction times (first peak: $\beta_{mean}=7.33$, t(19)=0.16; second peak: $\beta_{mean}=35.92$, t(19)=0.70) or the probability to make a correct choice (first peak: $\beta_{mean}=-0.12$, t(19)=−0.16; second peak: $\beta_{mean}=-0.17$, t(19)=−0.27).

## Data Availability

• Data to reproduce the results can be found at https://github.com/schwartenbeckph/Generative-Replay. It is legally unclear whether individual fMRI data are considered anonymous, but they can be shared upon reasonable request and a signed agreement that no attempt will be made to de-anonymise the data or share them onwards. • All original code has been deposited at https://github.com/schwartenbeckph/Generative-Replay and is publicly available as of the date of publication. DOIs are listed in the key resources table. • Any additional information required to reanalyze the data reported in this paper is available from the lead contact upon request.
